# Prophylactic and therapeutic potential of magnolol-loaded PLGA-PEG nanoparticles in a chronic murine model of allergic asthma

**DOI:** 10.3389/fbioe.2023.1182080

**Published:** 2023-05-04

**Authors:** Junyi Wang, Mo Xian, Hui Cao, Lei Wu, Libo Zhou, Yihe Ma, Long Fan, Lin Lin, Guoping Li, Qinmiao Huang, Shau-Ku Huang, Xiaojun Xiao

**Affiliations:** ^1^ Shenzhen Key Laboratory of Allergy and Immunology, Guangdong Provincial Standardization Allergen Engineering Research Center, State Key Laboratory of Respiratory Disease Shenzhen University Division, Institute of Allergy and Immunology, Shenzhen University School of Medicine, Shenzhen, China; ^2^ Laboratory of Allergy and Precision Medicine, Department of Pulmonary and Critical Care Medicine, Chengdu Institute of Respiratory Health, The Third People’s Hospital of Chengdu, Affiliated Hospital of Southwest Jiaotong University, Chengdu, China; ^3^ Department of Allergy and Clinical Immunology, State Key Laboratory of Respiratory Disease, The First Affiliated Hospital of Guangzhou Medical University, Guangzhou, China; ^4^ Department of Pulmonary and Critical Care Medicine, Third Affiliated Hospital of Shenzhen University, Shenzhen, China; ^5^ State Key Laboratory of Dampness Syndrome of Chinese Medicine, The Second Affiliated Hospital of Guangzhou University of Chinese Medicine, Guangzhou, China; ^6^ Guangdong Provincial Hospital of Chinese Medicine, Guangdong Provincial Academy of Chinese Medical Sciences, Guangzhou, China; ^7^ Guangdong-Hong Kong-Macau Joint Lab on Chinese Medicine and Immune Disease Research, Guangzhou, China; ^8^ Department of Urology, The First Affiliated Hospital of Nanchang University, Nanchang, China

**Keywords:** asthma, murine model, magnolol, nanoparticles, anti-allergic drugs

## Abstract

Magnolol is a chemically defined and active polyphenol extracted from magnolia plants possessing anti-allergic activity, but its low solubility and rapid metabolism dramatically hinder its clinical application. To improve the therapeutic effects, magnolol-encapsulated polymeric poly (DL-lactide-co-glycolide)–poly (ethylene glycol) (PLGA-PEG) nanoparticles were constructed and characterized. The prophylactic and therapeutic efficacy in a chronic murine model of OVA-induced asthma and the mechanisms were investigated. The results showed that administration of magnolol-loaded PLGA-PEG nanoparticles significantly reduced airway hyperresponsiveness, lung tissue eosinophil infiltration, and levels of IL-4, IL-13, TGF-β_1_, IL-17A, and allergen-specific IgE and IgG_1_ in OVA-exposed mice compared to their empty nanoparticles-treated mouse counterparts. Magnolol-loaded PLGA-PEG nanoparticles also significantly prevented mouse chronic allergic airway mucus overproduction and collagen deposition. Moreover, magnolol-encapsulated PLGA-PEG nanoparticles showed better therapeutic effects on suppressing allergen-induced airway hyperactivity, airway eosinophilic inflammation, airway collagen deposition, and airway mucus hypersecretion, as compared with magnolol-encapsulated poly (lactic-co-glycolic acid) (PLGA) nanoparticles or magnolol alone. These data demonstrate the protective effect of magnolol-loaded PLGA-PEG nanoparticles against the development of allergic phenotypes, implicating its potential usefulness for the asthma treatment.

## Introduction

Asthma is a common chronic inflammatory disease affecting an estimated 262 million people in different countries (Asher et al., 2020). Airway hyperresponsiveness (AHR), type 2 inflammation, airway remodeling, and mucus hypersecretion are hallmarks of asthmatic processes (Holgate et al., 2015). These pathological changes cause asthma symptoms, which can be any combination of coughing, wheezing, shortness of breath, and chest tightness. To date, glucocorticoids still represent the mainstay of asthma control, but these drugs come with side effects, including suppression of the host defense and metabolic impairments, particularly with systematic use (Okwuofu et al., 2022).

Magnolol (5,5′-diallyl-2,2′-dihydroxybiphenyl) is an active polyphenol extracted from *Magnolia officinalis*, which is a traditional Chinese medicine with a long history of application to prevent cardiovascular and cerebrovascular diseases, treat depression and anxiety, and relieve asthma and cough (Yang et al., 2023). Pharmaceutically, magnolol has anti-oxidant, anti-inflammatory, anti-microbial, anti-tumor, cardiovascular, and neural protective properties (Mainardi et al., 2009). Recently, magnolol has been found to have anti-allergic effects on allergic rhinitis via the inhibition of ORAI1 (calcium release-activated calcium channel protein 1) and ANO1 (a calcium-activated anion channel 1) channels (Phan et al., 2022). Moreover, magnolol exerts anti-asthmatic effects via its ability to modulate Th1/Th2/Th17 cytokines in ovalbumin-sensitized asthmatic mice (Huang et al., 2019). Its isomer, honokiol, has also been shown to alleviate the inflammatory processes contributing to asthma (Munroe et al., 2010). However, the low water solubility and bioavailability and the rapid metabolism of magnolol dramatically limit its clinical application (Tang et al., 2018). Thus, a critical question regarding magnolol usage is how bioavailability and stability can be improved.

Biodegradable polymeric nanoparticles have various advantages, such as high biocompatibility and biosafety, in potentiating the efficacy of drugs (Shao et al., 2022). Although poly (lactic-co-glycolic acid) (PLGA), a synthetic polymeric material certified by the FDA, is widely used to prevent clinical drugs from degradation, it suffers from an array of shortcomings, including low encapsulation efficiency of polar drugs and high capture rate by the reticuloendothelial system due to its hydrophobicity (Younis et al., 2022). Poly (ethylene glycol) (PEG) has the two affinity characteristics of dissolving in water and organic solvents, meaning it shows a potential to promote the hydrophilicity, drug encapsulation efficiency, and blood circulation time of PLGA (Padin-Gonzalez et al., 2022). Previously, we found that the *Ambrosia artemisiifolia* allergen Amb a 1-loaded poly (DL-lactide-co-glycolide)-poly (ethylene glycol) (PLGA-PEG) nanoparticles have an immunotherapeutic effect on allergic conjunctivitis in mice (Cao et al., 2022). In this study, we hypothesized that magnolol-loaded PLGA-PEG nanoparticles could be effective in attenuating asthma phenotypes in a chronic murine model.

## Materials and methods

### Animals

Female BALB/c mice (specific pathogen-free grade, body weight 16–22 g, 6–8 weeks old) were purchased from the Animal Center of Guangdong Province and maintained under specific pathogen-free conditions in the Animal Experimental Center of Shenzhen University. All experiments were approved by the Animal Ethic Committee at Shenzhen University. The experiments were carried out following the Institutional Guidelines for the Care and Use of Laboratory Animals.

### Preparation of magnolol-loaded nanoparticles

The nanoparticles were prepared by the emulsification–solvent evaporation method (Paswan and Saini, 2017). Briefly, 1 mg magnolol (MCE, United States) and 4 mg PLGA-PEG/PLGA (Merck, Germany) were dissolved in a 2 mL mixture of dichloromethane and ethanol (4:1, v/v) and injected into 4 mL 1.5% aqueous PVA solution. The mixture was homogenized with a probe-ultrasound machine (VCX750, Sonics, United States) for 5 min in an ice bath and stirred uncovered for 12 h at room temperature to volatilize the organic solvent completely. Then, the nanoparticle suspension was filtered by a 0.22 μm microporous membrane.

### Characterization of magnolol-loaded nanoparticles

The zeta potential of the nanoparticles was measured by the Zetasizer Ultra instrument (Malvern, United Kingdom). After the nanoparticles were diluted with ultrapure water, the size distribution of the nanoparticles was also measured by this instrument. After the nanoparticles were fixed on the stub with double-sided adhesive tape, they were coated with a platinum layer by an automatic fine platinum coater (JFC-1300, JEOL) for 1 min, and then their morphology was observed by a field-emission scanning electron microscope (FESEM). For the measurement of encapsulation efficiency, the prepared nanoparticle suspension was added into an ultrafiltration tube with a molecular weight cut-off of 3 kD, centrifuged at 4,000 rpm for 30 min to separate the magnolol that was not coated by nanoparticles, and the concentration of magnolol was measured by HPLC. The following formula was used to calculate the encapsulation efficiency (EE): EE (%) = [(mtotal-mfree)/mtotal] × 100%. Mtotal is the concentration of magnolol in total suspension, and Mfree is the concentration of magnolol in the ultrafiltrate.

### Sensitization, challenge, and administration protocols

As shown schematically in Figure 2A, the mice were immunized intraperitoneally with 10 μg OVA (Sigma-Aldrich, United States) adsorbed to 1 mg of alum (Thermo Scientific, United States) on days 0 and 14 (Royce et al., 2013; Wang et al., 2017a). At day 21, the mice were challenged with intranasal instillation of 20 μg OVA in a 20 μL PBS. The OVA challenge was performed three times per week for 6 weeks. Twenty mg/kg of magnolol (Herbpurify, China) and magnolol-loaded nanoparticles were administered intraperitoneally 24 h before the first OVA challenge and 2 h before each of the remaining OVA challenges (Tian et al., 2018; Huang et al., 2019). PBS was used as an experimental control.

### AHR assay

AHR was measured with the Buxco whole-body plethysmography (WBP) system (Buxco Research Company, United States) in response to inhaled methacholine. After 24 h of the last OVA challenge, the mice were monitored for about 10 min in the chamber until their breathing became stable. After a baseline recording for 5 min, the responses were assessed for 5 min after the inhaling of different concentrations of atomized methacholine solutions (0, 6.25, 12.5, 25, 50, and 100 mg/mL). In order to allow the respiratory intensity to get back to the baseline, an interval of 5 min was given between each test. AHR was expressed as enhanced pause (Penh), as described in detail previously (Wang et al., 2017b).

### Bronchoalveolar lavage fluid (BALF) collection

The mice were subjected to tracheotomy and intubation after euthanasia via carbon dioxide inhalation. A five-bouts lavage with 0.8 mL PBS was performed on each mouse three times. A total volume of about 2 mL BALF per mouse (recovery rate >80%) was collected and centrifuged at 4°C, 1500 rpm for 10 min. The supernatant was used for cytokine determination, and the precipitate was resuspended and stained for inflammatory cell differential counting via Liu’s staining method, following the instructions of the manufacturer (Baso, China).

### Lung histological staining

The lungs were immediately removed after sacrifice, fixed in 4% paraformaldehyde, and embedded in paraffin. Lung sections (4 µm) were stained with hematoxylin–eosin (H&E), periodic acid–Schiff (PAS), and Masson’s trichrome methods. The degree of inflammatory infiltration on H&E staining sections was scored using previously described methods (Wang et al., 2017b). PAS staining was used to identify the mucus-producing goblet cells in the airway mucosa (Wang et al., 2017b). Masson’s trichrome staining was used to detect peri-bronchial collagen deposition. A score ranging from 0 to 3 was applied to each observed bronchus, with an approximate total of 10 areas being scored (Li et al., 2013).

### Quantitative reverse transcription PCR (qRT-PCR)

The total RNA was extracted from the lung tissues with TRIzol Reagent (Thermo Scientific, United States), as recommended by the manufacturer. A total of 1.5 μg of total RNA preparation was reverse transcribed using a cDNA synthesis kit (RevertAid First Strand cDNA Synthesis Kit, Thermo Scientific, United States). cDNA was 1/5 diluted, and 5 μL was used as a template in a 50 μL SYBR-Green PCR reaction system, according to the manufacturer’s instruction (iQ™ SYBR^®^ Green, Bio-Rad, United States). β-actin premier (sense, 5′-CAT​CCG​TAA​AGA​CCT​CTA​TGC​CAA​C-3’; antisense, 5′-ATG​GAG​CCA​CCG​ATC​CAC​A-3′), *Muc5ac* premier (sense, 5′-CTG​TGA​CAT​TAT​CCC​ATA​AGC​CC-3’; antisense, 5′-ACC​GAT​CCC​GCC​CAG​TGA​CA-3′), and *Col1a1* premier (sense, 5′-TGT​TCG​TGG​TTC​TCA​GGG​TAG-3’; antisense, 5′-TTG​TCG​TAG​CAG​GGT​TCT​TTC-3′) were synthesized by Sangon Biotech (Shanghai, China). Specificity was controlled by the omission of the template or the reverse transcription. All the samples were run in triplicate, and the qRT-PCR results were obtained using the 2^−△△Ct^ method and were normalized to β-actin.

### Enzyme-linked immunosorbent assay (ELISA)

The levels of IL-4, IL-13, IL-17A, and TGF-β_1_ in the BALF were determined by ELISA with commercial kits (eBioscience, United States), in accordance with instructions of the manufacturer. OVA-specific IgE (sIgE) and OVA-specific IgG_1_ (sIgG_1_) were measured by indirect ELISA (Tian et al., 2018). Briefly, the 96-well plates were coated with 100 ng OVA overnight at 4°C, blocked at room temperature for 1 h, and 100 μL murine serum (diluted 5 times) was added to each well for 2 h. Peroxidase-conjugated rat anti-mouse IgE and IgG_1_ (1:2000 dilution, Southern Biotech, United States) were added to each well for 1 h (37°C), and then 100 μL/well tetramethylbenzidine was added to develop. After being stopped by 2 M H_2_SO_4_ (50 μL/well), the results were measured by an absorbance microplate reader (BioTek, United States) at 450 nm.

### Statistical methodology

The data were represented as means ± SD from at least three independent experiments. Statistical analyses were performed using a non-paired *t*-test for comparing two groups, and multiple comparisons were carried out with ANOVA, followed by Dunnett’s test or the Bonferroni test for those with more than two groups. *p* < 0.05 was considered statistically significant. All data were analyzed by the SPSS 21.0 software.

## Results

### Characterization of magnolol-loaded PLGA-PEG nanoparticles

**FIGURE 1:**
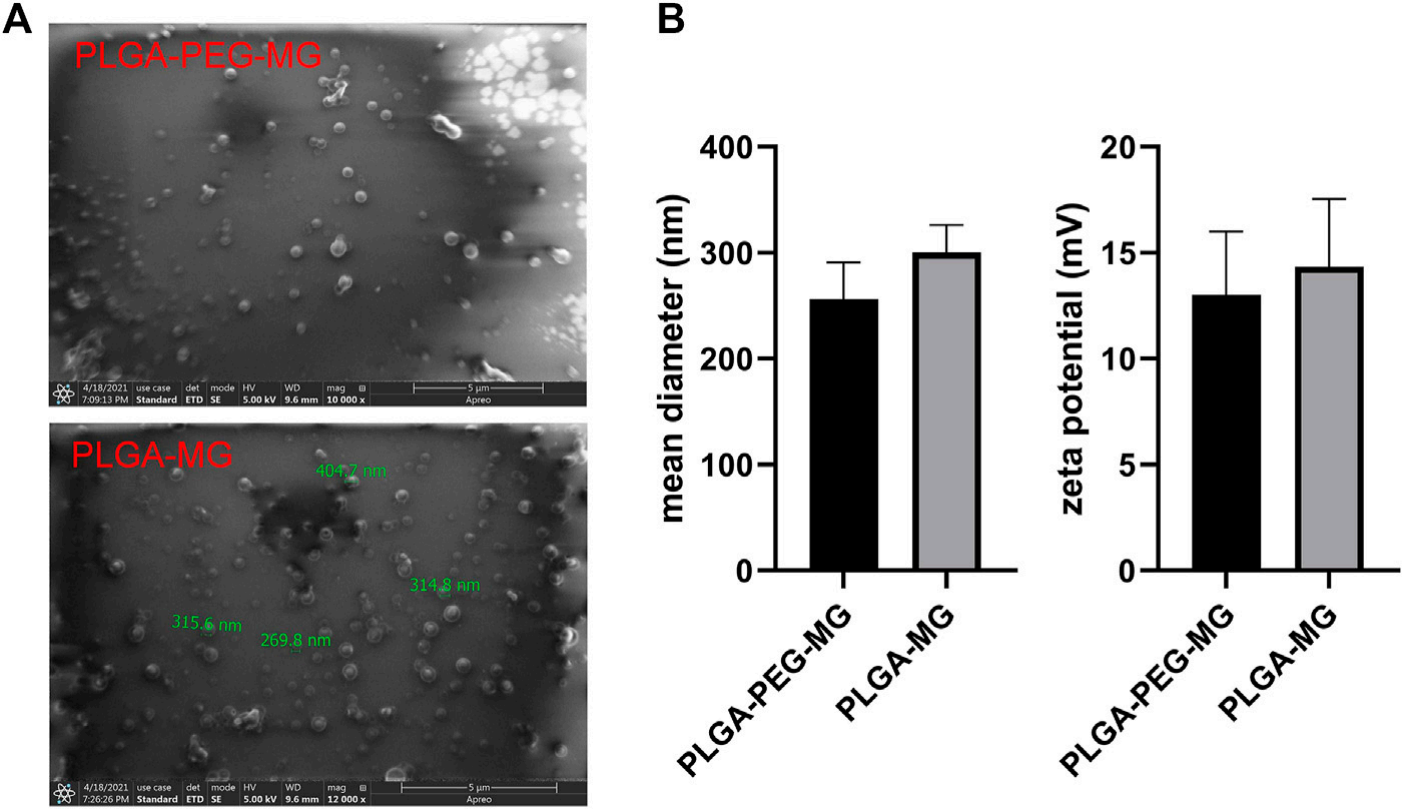
Characterization of magnolol-loaded PLGA-PEG nanoparticles. **(A)** Field emission scanning electron microscopic image. **(B)** Mean diameter and zeta potential of the two nanoparticle preparations (*n* = 3). PLGA-MG: magnolol-loaded PLGA nanoparticle; PLGA-PEG-MG: magnolol-loaded PLGA-PEG nanoparticle.

We prepared the magnolol-loaded nanoparticles with PLGA-PEG and PLGA as carriers (Figure 1A), and their average particle size and zeta potential are shown in Figure 1B. The average particle size and zeta potential of PLGA-PEG-magnolol were 230 nm and 12.5 mV, respectively. The average particle size and zeta potential of PLGA-magnolol were 290 nm and 14.5 mV, respectively. The encapsulation efficiency of PLGA-PEG to magnolol was 90.5% ± 5%, and PLGA to magnolol was 90.2% ± 4%.

### Magnolol-loaded PLGA-PEG nanoparticles migrate allergen-induced AHR

**FIGURE 2:**
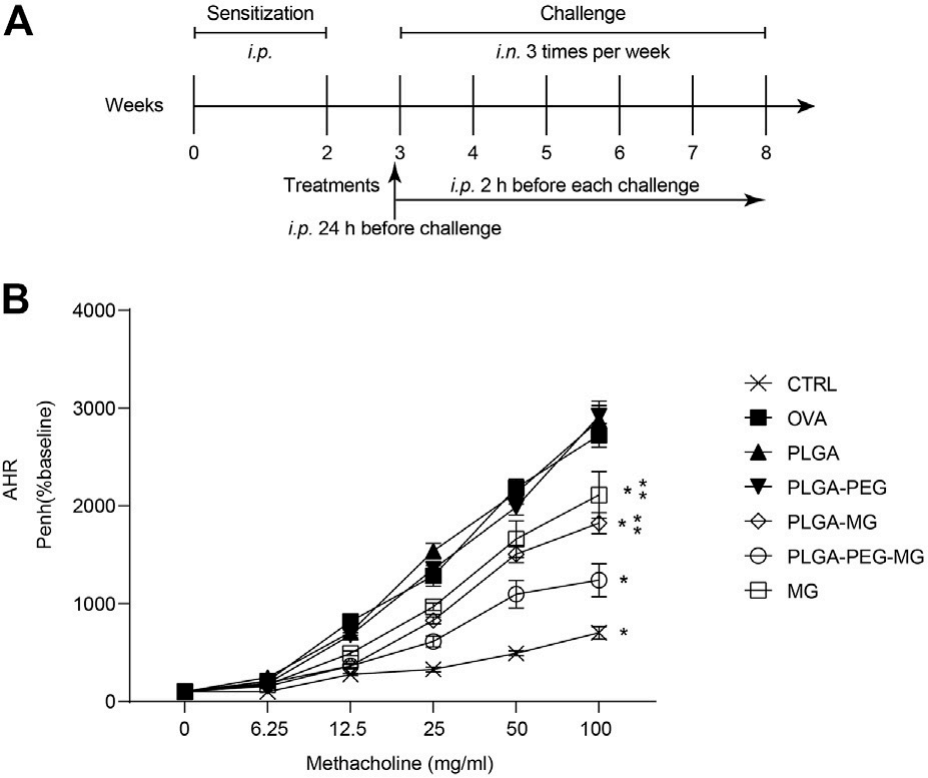
Magnolol-loaded PLGA-PEG nanoparticles migrate allergen-induced AHR. **(A)** Schematic representation of the mouse model. **(B)** The data are shown as means ± SD from four individual mice. CTRL: saline control group; OVA: OVA model group; PLGA: drug-free PLGA nanoparticle group; PLAG-PEG: drug-free PLGA-PEG nanoparticle group; PLGA-MG: magnolol-loaded PLGA nanoparticle group; PLGA-PEG-MG: magnolol-loaded PLGA-PEG nanoparticle group; MG: magnolol group. *p < 0.05 versus OVA group, **p < 0.05 versus magnolol-loaded PLGA-PEG nanoparticle group.

AHR is a fundamental hallmark of asthma (Holgate et al., 2015). In order to investigate whether the magnolol-loaded nanoparticles improved lung function in asthmatic mice, we assessed AHR by methacholine exposure 24 h after the last OVA challenge (Figure 2B). The enhanced pause (Penh) scores of the OVA model (OVA) group and the two drug-free vehicle nanoparticle (PLGA and PLAG-PEG) groups were increased in a dose-dependent manner as compared to the saline control (CTRL) group. However, AHR was significantly inhibited in the magnolol-treated (MG) group and the two magnolol-loaded nanoparticles (PLGA-MG and PLGA-PEG-MG) groups. Moreover, among the three groups, the PLGA-PEG-MG group exhibited the lowest Penh levels. These data show that magnolol-loaded PLGA-PEG nanoparticles markedly inhibit OVA-induced AHR.

### Magnolol-loaded PLGA-PEG nanoparticles alleviate OVA-induced lung inflammation

**FIGURE 3:**
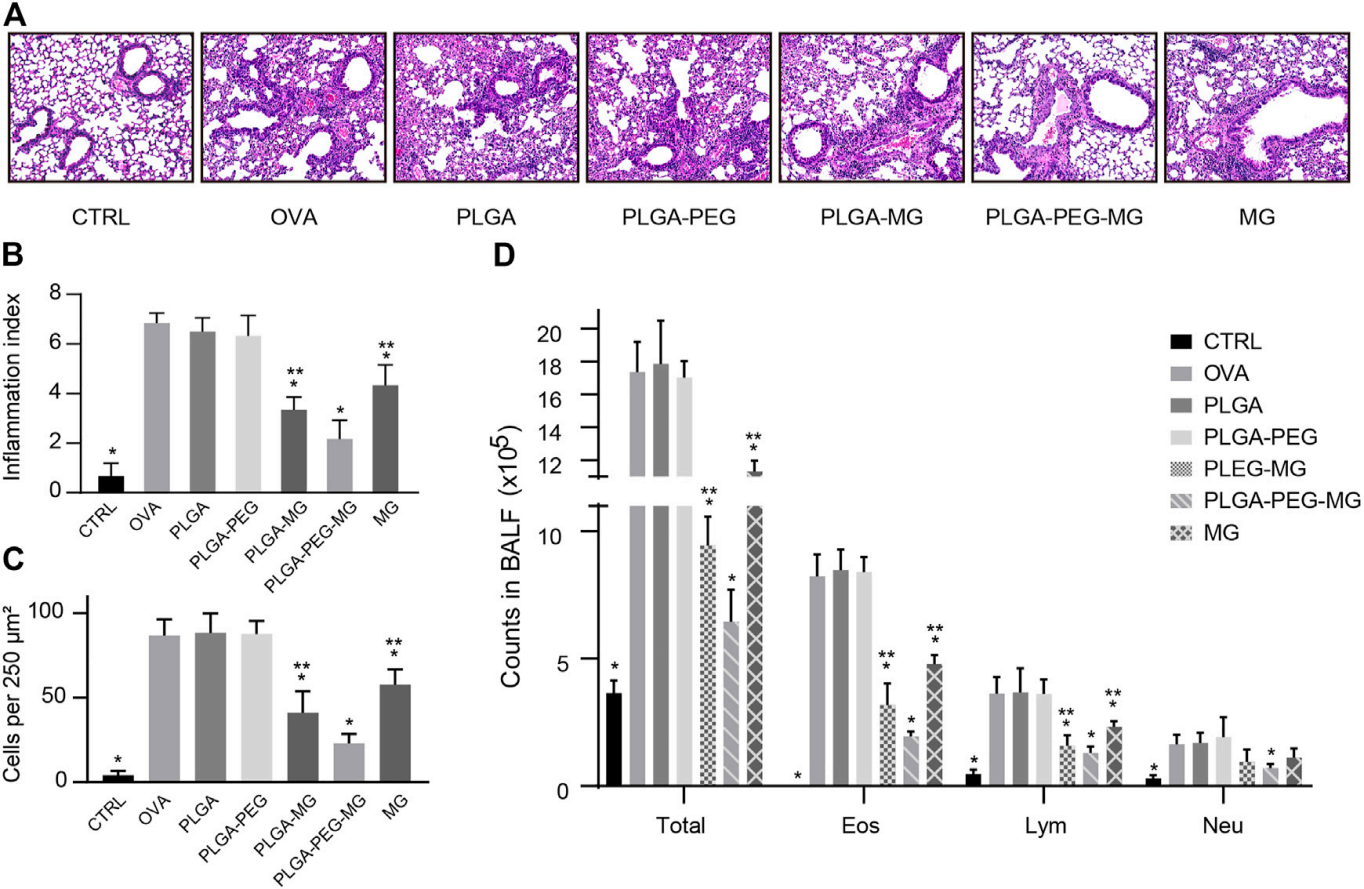
Magnolol-loaded PLGA-PEG nanoparticles alleviate OVA-induced lung inflammation. **(A)** Representative histological images of lungs by H&E staining (Magnification: ×200). **(B,C)** Inflammation scores **(B)** and numbers of inflammatory cells **(C)** estimated from lung tissues with H&E staining. **(D)** Counts of total cells, eosinophils, lymphocytes, and neutrophils in the BALF. The data are shown as means ± SD from six individual mice. CTRL: saline control group; OVA: OVA model group; PLGA: drug-free PLGA nanoparticle group; PLAG-PEG: drug-free PLGA-PEG nanoparticle group; PLGA-MG: magnolol-loaded PLGA nanoparticle group; PLGA-PEG-MG: magnolol-loaded PLGA-PEG nanoparticle group; MG: magnolol group. *p < 0.05 versus OVA group, **p < 0.05 versus magnolol-loaded PLGA-PEG nanoparticle group.

The lung tissues and bronchoalveolar lavage fluid (BALF) were collected 24 h after the last OVA exposure. To evaluate the inflammatory infiltration, the lung tissues were stained with H&E (Figure 3A) and scored in a blinded fashion (Figure 3B). The number of infiltrating inflammatory cells was also evaluated on the HE-stained sections (Figure 3C). Compared to the OVA group, the inflammatory cell infiltration in the lungs was reduced in PLGA-PEG-MG group, PLGA-MG group, and MG group. The PLGA-PEG-MG group had less inflammatory infiltration in the lungs compared to the PLGA-MG group and MG group. In the meantime, the counts of total cells, eosinophils, and lymphocytes in the BALF upon OVA exposure were also decreased significantly after the treatments of magnolol-loaded nanoparticles and magnolol alone (Figure 3D). Of these, the treatment of the magnolol-loaded PLGA-PEG nanoparticles exhibited the most pronounced reduction in inflammatory cell numbers. However, the mice treated with drug-free nanoparticles did not display the similar reduction of inflammatory cell infiltration both in the lungs and BALF. These findings indicate that magnolol-loaded PLGA-PEG nanoparticles attenuate OVA-induced lung inflammation.

### Magnolol-loaded PLGA-PEG nanoparticles inhibit OVA-induced airway mucus hypersecretion

**FIGURE 4:**
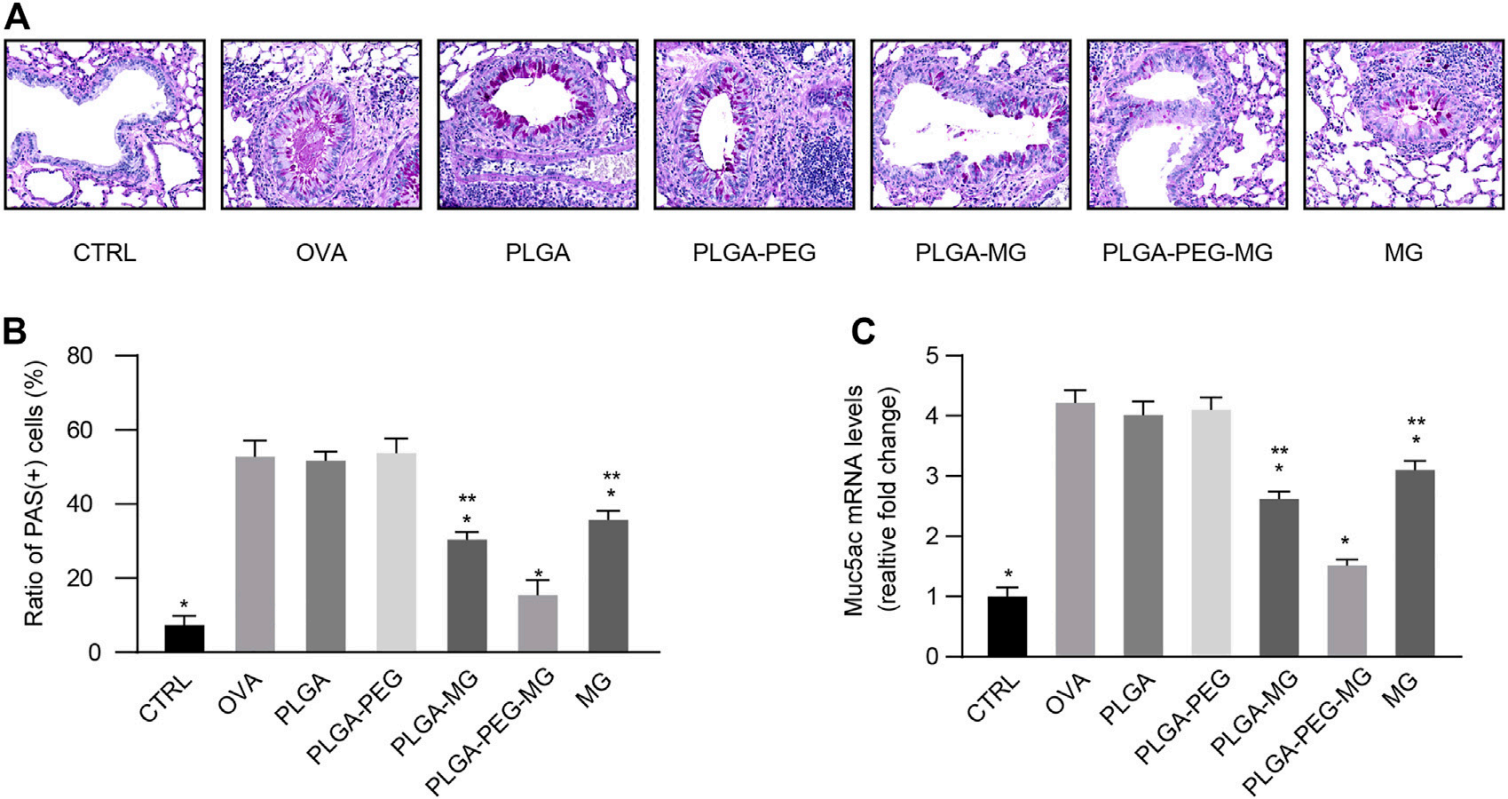
Magnolol-loaded PLGA-PEG nanoparticles inhibit OVA-induced airway mucus hypersecretion. **(A)** Representative histological images of lungs by PAS staining (Magnification: ×400). **(B)** Scoring for PAS-positive mucus-producing cells from lung tissues. **(C)** qRT-PCR determination of mRNA levels for *Muc5ac*. The data are shown as means ± SD from three individual mice. CTRL: saline control group; OVA: OVA model group; PLGA: drug-free PLGA nanoparticle group; PLAG-PEG: drug-free PLGA-PEG nanoparticle group; PLGA-MG: magnolol-loaded PLGA nanoparticle group; PLGA-PEG-MG: magnolol-loaded PLGA-PEG nanoparticle group; MG: magnolol group. *p < 0.05 versus OVA group, **p < 0.05 versus magnolol-loaded PLGA-PEG nanoparticle group.

Excessive mucus in asthma obstructs airflow, leading to severe and potentially fatal outcomes (Holgate et al., 2015). A previous study suggested that chronic allergen exposure promotes goblet cell hyperplasia and mucin overproduction (Southam et al., 2008). We first observed the impact of the magnolol-loaded nanoparticles on aspects of goblet cell hyperplasia by PAS staining (Figures 4A, B). The PAS-positive cells were readily seen in the OVA group and the two drug-free nanoparticle groups, but they were much less apparent in the PLGA-PEG-MG group, PLGA-MG group, and MG group. Furthermore, the number of positively stained cells was significantly lower in the PLGA-PEG-MG group than those of the PLGA-MG group and MG group. We then determined the *Muc5ac* expression levels in the lungs via qRT-PCR (Figure 4C). Consistently, the expression of *Muc5ac* induced by OVA exposure was dramatically inhibited by treatments with magnolol-loaded nanoparticles and magnolol alone. In particular, the PLGA-PEG-MG group had the lowest expression of *Muc5ac*. Treatment with drug-free nanoparticles had no effect on the PAS-positive cell number and *Muc5ac* expression. These results suggest that magnolol-loaded PLGA-PEG nanoparticles reduce OVA-induced goblet cell hyperplasia and mucin hyperproduction.

### Magnolol-loaded PLGA-PEG nanoparticles suppress OVA-induced peri-bronchial collagen deposition

**FIGURE 5:**
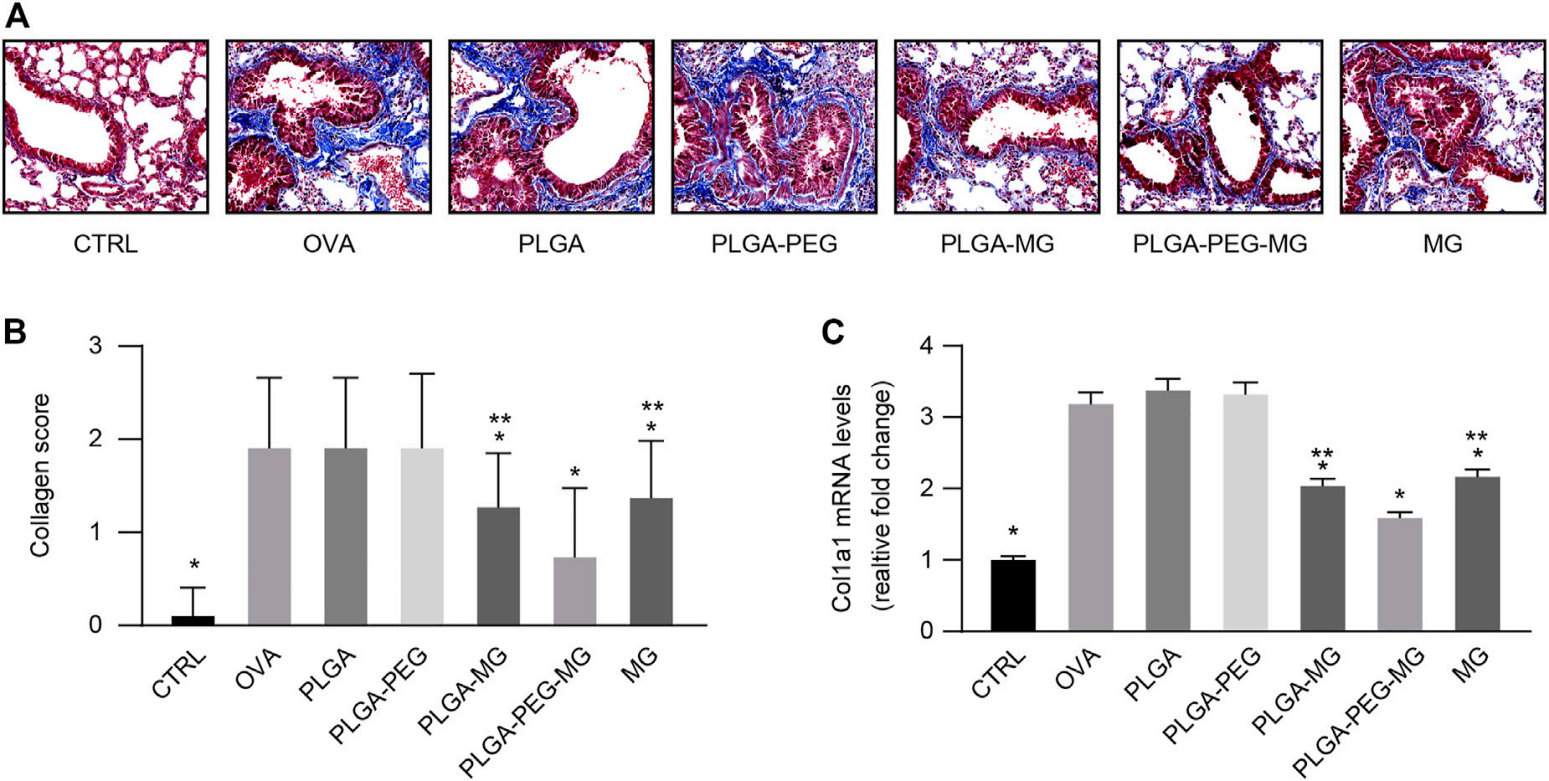
Magnolol-loaded PLGA-PEG nanoparticles suppress OVA-induced peri-bronchial collagen deposition. **(A)** Representative histological images of lungs by Masson’s trichrome staining (Magnification: ×400). **(B)** Peri-bronchial collagen deposition scores estimated from lung tissues with Masson’s trichrome staining. **(C)** qRT-PCR determination of mRNA levels for *Col1a1*. The data are shown as means ± SD from three individual mice. CTRL: saline control group; OVA: OVA model group; PLGA: drug-free PLGA nanoparticle group; PLAG-PEG: drug-free PLGA-PEG nanoparticle group; PLGA-MG: magnolol-loaded PLGA nanoparticle group; PLGA-PEG-MG: magnolol-loaded PLGA-PEG nanoparticle group; MG: magnolol group. *p < 0.05 versus OVA group, **p < 0.05 versus magnolol-loaded PLGA-PEG nanoparticle group.

Airway remodeling contributes to the progressive loss of lung function in asthma (Li et al., 2013). Collagen deposition is an acknowledged feature of airway remodeling during asthma (Ramis et al., 2022). Using Masson’s trichrome staining (Figures 5A, B), we found that chronic OVA exposure increased the deposition of collagen around the airways, and these increases were attenuated in the PLGA-PEG-MG group, PLGA-MG group, and MG group. Furthermore, the PLGA-PEG-MG group had the greatest inhibition in the three treatments. Meanwhile, the expression of *Col1a1* was evaluated by qRT-PCR (Figure 5C). Consistently, the treatments of magnolol-loaded nanoparticles and magnolol alone suppressed the enhanced levels of *Col1a1* in OVA-exposed mice. Compared with the magnolol-loaded PLGA nanoparticles and magnolol-treated mice, the levels of *Col1a1* mRNA were significantly decreased in the magnolol-loaded PLGA-PEG nanoparticles-treated mice. In addition, no significant inhibition of either collagen deposition or *Col1a1* expression was observed in drug-free nanoparticles-treated mice. These results demonstrate that PLGA-PEG nanoparticles containing magnolol suppress collagen deposition induced by chronic OVA exposure.

### Magnolol-loaded PLGA-PEG nanoparticles reduce OVA-induced specific immunoglobulin levels in serum

**FIGURE 6:**
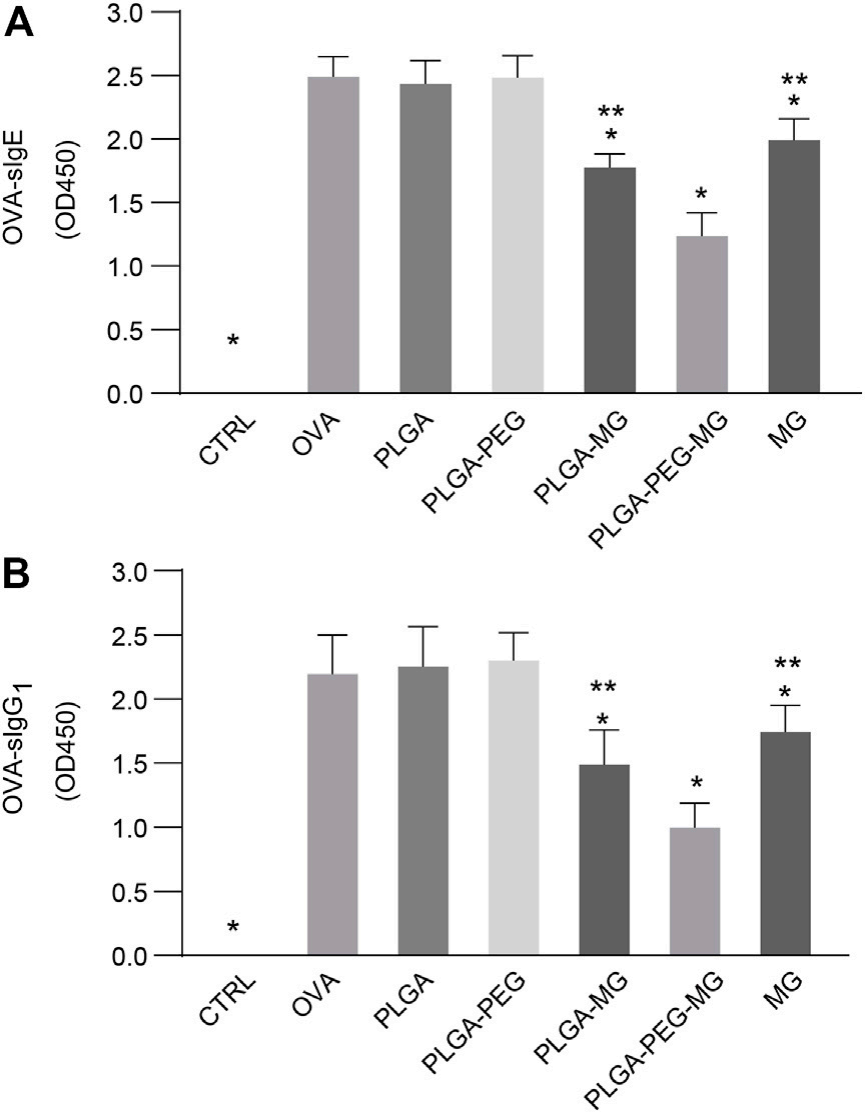
Magnolol-loaded PLGA-PEG nanoparticles reduce OVA-induced specific immunoglobulin levels in serum. **(A,B)** OVA-specific IgE **(A)** and OVA-specific IgG_1_ **(B)** levels in serum by indirect ELISA. The data are shown as means ± SD from six individual mice. CTRL: saline control group; OVA: OVA model group; PLGA: drug-free PLGA nanoparticle group; PLAG-PEG: drug-free PLGA-PEG nanoparticle group; PLGA-MG: magnolol-loaded PLGA nanoparticle group; PLGA-PEG-MG: magnolol-loaded PLGA-PEG nanoparticle group; MG: magnolol group. *p < 0.05 versus OVA group, **p < 0.05 versus magnolol-loaded PLGA-PEG nanoparticle group.

Serum was collected 24 h after the last OVA challenge, and then OVA-specific IgE (sIgE) (Figure 6A) and OVA-specific IgG1 (sIgG_1_) (Figure 6B) levels were determined via ELISA. OVA exposure with or without administration of empty nanoparticles led to a marked elevation in the levels of sIgE and sIgG_1_ as compared with the control mice. These elevations were suppressed by the magnolol-loaded PLGA-PEG nanoparticles, magnolol-loaded PLGA nanoparticles, and magnolol. The suppression of the magnolol-loaded PLGA-PEG nanoparticles was greater than those of the magnolol-loaded PLGA nanoparticles and magnolol. These findings indicate that magnolol-loaded PLGA-PEG nanoparticles decrease OVA-induced sIgE and sIgG_1_ secretion in serum.

### Magnolol-loaded PLGA-PEG nanoparticles regulate OVA-induced cytokine production

**FIGURE 7:**
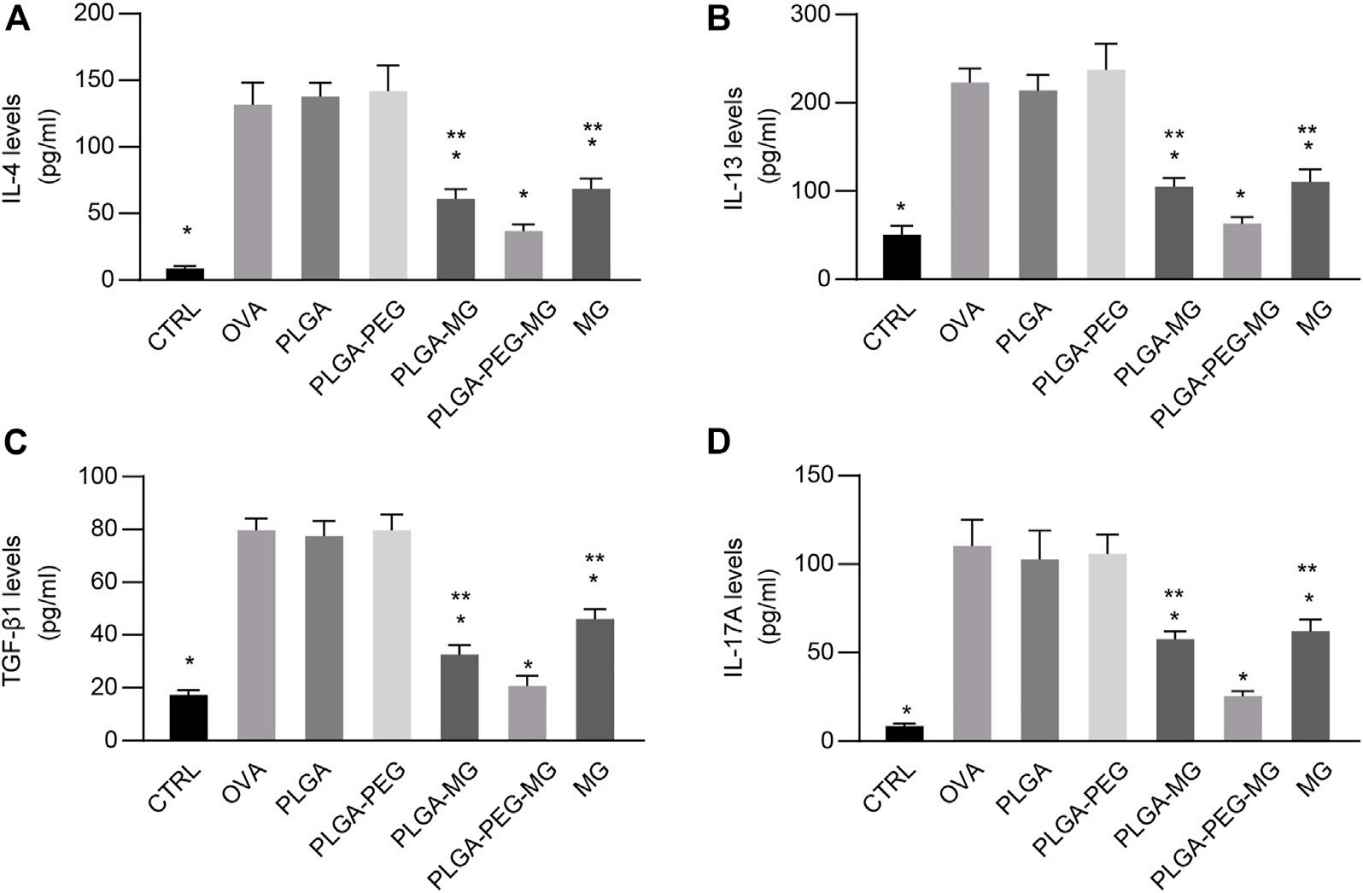
Magnolol-loaded PLGA-PEG nanoparticles regulate OVA-induced cytokine production. **(A–D)** IL-4 **(A)**, IL-13 **(B)**, TGF-β_1_ **(C)**, and IL-17A **(D)** levels in the BALF by ELISA. The data are shown as means ± SD from six individual mice. CTRL: saline control group; OVA: OVA model group; PLGA: drug-free PLGA nanoparticle group; PLAG-PEG: drug-free PLGA-PEG nanoparticle group; PLGA-MG: magnolol-loaded PLGA nanoparticle group; PLGA-PEG-MG: magnolol-loaded PLGA-PEG nanoparticle group; MG: magnolol group. *p < 0.05 versus OVA group, **p < 0.05 versus magnolol-loaded PLGA-PEG nanoparticle group.

The levels of cytokines in the BALF were measured with ELISA. We observed a robust reduction of IL-4 (Figure 7A), IL-13 (Figure 7B), TGF-β_1 (_ Figure 7C), and IL-17A (Figure 7D) levels in the magnolol-loaded nanoparticles and magnolol-treated animals compared to the OVA-exposed animals. Meanwhile, the concentrations of IL-4, IL-13, TGF-β_1_, and IL-17A in the PLGA-PEG-MG mice were significantly lower compared to the PLGA-MG mice and MG mice. These results suggest that magnolol-loaded PLGA-PEG nanoparticles decrease IL-4, IL-13, TGF-β_1_, and IL-17A expression during OVA-induced airway inflammation.

## Discussion

Here, we successfully constructed magnolol-loaded PLGA-PEG nanoparticles through the w/o/w double emulsion–solvent evaporation method. The results showed that the mean hydrodynamic size of the magnolol-loaded PLGA-PEG nanoparticles was about 200.1–260.2 nm in diameter. This property is excellent for passive targeting to the sites of inflammation. Moreover, the size distribution of the magnolol-loaded nanoparticles was comparatively narrow, which is conducive for magnolol delivery.

Allergic airway inflammation is mainly orchestrated by type 2 cytokines such as IL-4 and IL-13, and it is marked by the massive infiltration of eosinophils (Holgate et al., 2015). Activation of the IL-4/IL-13 pathway promotes profound airway hyperresponsiveness (Manson et al., 2020). Inhibition of IL-4 and IL-13 significantly reduces IgE secretion in response to allergen challenge and further improves the infiltration of inflammatory cells in the airways (Castro et al., 2018). In an acute allergic mouse model, Huang et al. found that magnolol decreased cellular infiltration in the lungs, levels of IL-4 and IL-13 in the BALF, and sIgE levels in serum induced by an allergen (Huang et al., 2019). In this study, accompanied by high levels of IL-4, IL-13, and allergen-specific immunoglobulins, AHR and the number of eosinophils increased significantly in the lungs of chronic OVA-exposed mice. The treatments of magnolol-loaded PLGA-PEG nanoparticles, magnolol-loaded PLGA nanoparticles, and magnolol effectively mitigated these pathological changes. As expected, the magnolol-loaded PLGA-PEG nanoparticles successfully exhibited a more dramatic effect on the inhibition of AHR and type 2 cytokine-mediated airway inflammation, indicating a therapeutic potential of PLGA-PEG nanoparticles coated with anti-inflammatory drugs in allergic diseases.

Airway remodeling is a prominent clinical feature of severe asthma and may be responsible for the failure of standard anti-asthmatic therapy (Hirota and Martin, 2013). On the one hand, goblet cell hyperplasia and mucus hypersecretion are critical features of airway remodeling, leading to airway plugging and an increased risk of mortality (Boonpiyathad et al., 2019). Type 2 cytokines such as IL-13 promote hyperplasia of the goblet cell and hypersecretion of mucins including MUC5AC in asthmatics (Conde et al., 2021). Here, in addition to inhibition of type 2 cytokines, we also found that magnolol-loaded PLGA-PEG nanoparticles exhibited a greater improvement of goblet hyperplasia and MUC5AC overproduction than those of magnolol-loaded PLGA nanoparticles and magnolol. On the other hand, the chronic deposition of collagen fibers thickened the air–blood barrier, contributing to an irreversible decrement in lung function (Hirota and Martin, 2013). TGF-β_1_ is a member of the family of growth factors of crucial importance in fibrogenesis, and it plays an integral role in airway remodeling (Ojiaku et al., 2017). In the present study, peri-bronchial collagen deposition induced by allergen exposure was apparently decreased by the administration of magnolol-loaded nanoparticles and magnolol. The effects of magnolol-loaded PLGA-PEG nanoparticles were found to be much better than those of magnolol-loaded PLGA nanoparticles and magnolol alone. It was further confirmed by the observation that magnolol-loaded PLGA-PEG nanoparticles effectively downregulated TGF-β_1_ production in the lungs.

In addition, Th17 cells have emerged as an independent subset of CD4^+^ T-help cells. Th17 cells synthesizing IL-17A have been shown to play a crucial role in the induction of inflammatory diseases (Saviano et al., 2022). Accumulating evidence suggests that activation of the IL-17-producing cells is associated with the development of severe forms of asthma (Xie et al., 2022). A previous study showed that magnolol exerts anti-inflammatory effects when reducing the serum levels of IL-17 and IL-6 in a rat colitis model (Zhang et al., 2018). Another study showed that magnolol reduced the Th17 cell population and effectively modulated the JAK-STAT and Notch-1 signaling (Huang et al., 2019). Consistently, it had been also shown in the present study that magnolol-loaded PLGA-PEG nanoparticles remarkably suppressed the IL-17A expression in lungs and had a more potent effect than magnolol-loaded PLGA nanoparticles and magnolol alone.

In conclusion, we constructed PLGA-PEG nanoparticles as a magnolol delivery system and developed an OVA-induced chronic asthma murine model to evaluate the anti-inflammatory effects of these drug-loaded nanoparticles. Our results proved that magnolol-loaded PLGA-PEG nanoparticles could effectively suppress allergen-induced airway hyperactivity, airway eosinophilic inflammation, airway collagen deposition, and airway mucus hypersecretion. Furthermore, magnolol-loaded PLGA-PEG nanoparticles have a better therapeutic effect on OVA-induced asthmatic phenotypes than magnolol-loaded PLGA nanoparticles and magnolol alone, which may be due to their greater hydrophilicity, stability, and passive targeting effects. It should be acknowledged, however, that our study is limited by the OVA-induced model, which does not mimic the natural route of exposure to allergens. Future studies with more relevant allergic models, such as fungi and dust mites, are needed to validate and expand upon our findings.

## Data Availability

The raw data supporting the conclusion of this article will be made available by the authors, without undue reservation.
